# Prognostic prediction models for pregnancy complications in women with gestational diabetes: a protocol for systematic review, critical appraisal and meta-analysis

**DOI:** 10.1186/s13643-019-1151-0

**Published:** 2019-11-11

**Authors:** Shamil D. Cooray, Jacqueline A. Boyle, Georgia Soldatos, Lihini A. Wijeyaratne, Helena J. Teede

**Affiliations:** 1 Monash Centre for Health Research and Implementation, School of Public Health and Preventive Medicine, Monash University, Melbourne, Australia; 2 Diabetes and Vascular Medicine Unit, Monash Health, Melbourne, Australia; 3 Monash Women’s Program, Monash Health, Melbourne, Australia

**Keywords:** Gestational diabetes, Prediction model, Prognosis, Pregnancy complications, Macrosomia, Systematic review

## Abstract

**Background:**

Gestational diabetes (GDM) is increasingly common and has significant implications during pregnancy and for the long-term health of the mother and offspring. However, it is a heterogeneous condition with inter-related factors including ethnicity, body mass index and gestational weight gain significantly modifying the absolute risk of complications at an individual level. Predicting the risk of pregnancy complications for an individual woman with GDM presents a useful adjunct to therapeutic decision-making and patient education. Diagnostic prediction models for GDM are prevalent. In contrast, prediction models for risk of complications in those with GDM are relatively novel. This study will systematically review published prognostic prediction models for pregnancy complications in women with GDM, describe their characteristics, compare performance and assess methodological quality and applicability.

**Methods:**

Studies will be identified by searching MEDLINE and Embase electronic databases. Title and abstract screening, full-text review and data extraction will be completed independently by two reviewers. The included studies will be systematically assessed for risk of bias and applicability using appropriate tools designed for prediction modelling studies. Extracted data will be tabulated to facilitate qualitative comparison of published prediction models. Quantitative data on predictive performance of these models will be synthesised with meta-analyses if appropriate.

**Discussion:**

This review will identify and summarise all published prognostic prediction models for pregnancy complications in women with GDM. We will compare model performance across different settings and populations with meta-analysis if appropriate. This work will guide subsequent phases in the prognosis research framework: further model development, external validation and model updating, and impact assessment. The ultimate model will estimate the absolute risk of pregnancy complications for women with GDM and will be implemented into routine care as an evidence-based GDM complication risk prediction model. It is anticipated to offer value to women and their clinicians with individualised risk assessment and may assist decision-making. Ultimately, this systematic review is an important step towards a personalised risk-stratified model-of-care for GDM to allow preventative and therapeutic interventions for the maximal benefit to women and their offspring, whilst sparing expense and harm for those at low risk.

**Systematic review registration:**

PROSPERO registration number CRD42019115223

## Background

Gestational diabetes (GDM) is a state of carbohydrate intolerance resulting in hyperglycaemia that commences or is first recognised during pregnancy [1]. The prevalence of GDM is rising in the context of increasing maternal age and obesity, and introduction of new diagnostic criteria [2], representing a public health concern internationally [1]. Recent data found the prevalence of GDM to be approximately 13% [3], while in high-risk populations including some ethnic groups, prevalence is as high as 30% [4].

A diagnosis of GDM implies a state of glucometabolic dysfunction which is associated with an increased risk of pregnancy complications affecting the mother and fetus as well as having significant implications for the long-term health of the mother and offspring [5]. Landmark randomised controlled trials have demonstrated reduction in pregnancy risks with GDM management consisting of lifestyle modification and pharmacologic therapy, albeit based on less inclusive diagnostic criteria [6, 7].

New consensus-based diagnostic criteria were developed by the International Association of Diabetes in Pregnancy Study Groups (IADPSG) [8]. These criteria that generally include lower glucose cut-off levels are based on observational data [9] and remain highly controversial. These were endorsed by the World Health Organisation (WHO) and subsequently recommended by some [10] but not all professional societies [11]. Other societies acknowledge that different diagnostic criteria exist and that the optimal diagnostic strategies may vary depending on the characteristics of the local population [12–14].

Contemporary approaches to the diagnosis and management of GDM, regardless of diagnostic criteria, are glucocentric [15, 16]. Risks related to glucometabolic dysfunction are dichotomised on blood glucose levels into a binary yes/no GDM state. A one-size-fits-all model of intervention is then implemented, targeting glucose levels alone. This approach fails to appreciate the continuum of risk associated with blood glucose levels and the increased clinical heterogeneity of this condition, related primarily to ethnicity [17], increasing obesity [18, 19] and increasing excess gestational weight gain (GWG) [20]. These risk factors independently affect risk of diagnosis of GDM and short and long-term health outcomes for affected women [17–20]. This heterogeneity is likely explained by emerging physiologic data suggesting highly variable degrees of beta-cell function and insulin resistance amongst women diagnosed with this condition [21].

Limitations of this glucocentric approach and lack of risk stratification are evident from epidemiologic outcome data following the adoption of the new IADPSG diagnostic criteria. As expected, the application of these criteria have led to an increase in GDM incidence, with maternity centres reporting increases in the number of diagnoses by 28 to 74% [22–25]. This increasing incidence is most likely due to a change from a two- to one-step testing procedure and more inclusive blood glucose level criteria rather than changes in population characteristics. As such, a greater proportion of pregnancies are identified as being at high risk and treated with a package of care that includes additional education, lifestyle modification and pharmacologic therapy. However, intervening in a greater proportion of pregnancies has not led to an overall reduction in pregnancy complications [22, 26, 27] yet has increased the overall costs of GDM care [22] and psychosocial burden for affected women [28]. Therefore, there is a mandate to develop a more sophisticated prognosis and risk-stratified focused approach to GDM considering other relevant clinical factors driving adverse outcomes in addition to glycaemic measures.

A suitable and effective prognostic prediction model will allow calculation of the absolute risk of pregnancy complications for women with GDM who present for pregnancy-care based on their unique individual characteristics including BMI, GWG, ethnicity and obstetric history. Calculation of absolute risk of complications can then facilitate the development of stratified models-of-care that better meet the needs of women with this heterogeneous condition. This personalised medicine approach will facilitate the transition to a model-of-care where education, resources and specialist-care can be directed to those women most likely to benefit and sparing expense and unnecessary treatment from those who will not.

This review will answer the question, what prognostic prediction models have been developed for application to pregnancies affected by GDM to predict pregnancy complications and inform clinical therapeutic decision-making?

The objectives of this systematic review are

1. To identify existing prognostic prediction models for pregnancy complications in women with GDM;
2. To describe characteristics of the identified prognostic prediction models qualitatively;
3. To compare the performance of identified prognostic prediction models quantitatively across different settings and populations with the use of meta-analysis if appropriate;
4. To critically assess the conduct and reporting of methods of these prediction studies.

## Methods/design

This protocol is reported according to the Preferred Reporting Items for Systematic Reviews and Meta-Analyses Protocols (PRISMA-P) guideline [29] and the corresponding checklist used (Additional file 2). This systematic review protocol was registered on the PROSPERO international registry of systematic reviews on January 18, 2019 (CRD42019115223).

A systematic review of prediction modelling studies for pregnancy complications in women with GDM will be conducted to identify eligible studies published before December 2018. This review forms the foundations of a broader research program guided by the recommendations of The PROGnosis RESearch Strategy (PROGRESS) Partnership, an international, interdisciplinary collaboration that has published a framework to improve the standards of prognosis research to improve its translational impact. The framing of the review question (Table 1), study design, data extraction and appraisal will be guided by recent developments in prognosis research methodology, which seek to improve rigour and reproducibility. This includes the Cochrane Prognosis Methods Group Protocol Template [33], the TRIPOD statement (transparent reporting of a multivariable prediction model for individual prognosis or diagnosis) [34], the CHARMS checklist (checklist for critical Appraisal and data extraction for systematic reviews of prediction modelling studies) [30] and the PROBAST tool (prediction model risk of bias assessment) [35].

**Table 1:** Framing of this systematic review using key items identified by the CHARMS checklist [30]

Item	Comments
1. Prognostic versus diagnostic prediction model	Aim is to predict future events (prognostic prediction model)
2. Intended scope of the review	Models to inform clinicians’ therapeutic decision-making
3. Type of prediction modelling studies	All study types, i.e. Prediction model development studies with and without external validation and external model validation studies with or without model updating.
4. Target population to whom the prediction model applies	Pregnant women with gestational diabetes diagnosed according to each included study.
5. Outcome to be predicted	Pregnancy complications related to GDM affecting the mother (obstetric or maternal) or the child (fetal or neonatal). These complications were aligned with the standard outcomes agreed by consensus between review authors of Cochrane Pregnancy and Childbirth systematic reviews for the prevention and treatment of GDM and pre-existing diabetes [31, 32]. Complications potentially related to the treatment of GDM such as maternal hypoglycaemia and glycaemic control were not included. Gestational weight gain was not included as it is also likely to be a predictor of the outcomes of interest. Mother: • Hypertensive disorders of pregnancy (pregnancy-induced hypertension or pre-eclampsia) • Caesarean delivery • Maternal mortality • Placental abruption • Induction of labour • Perineal trauma • Postpartum haemorrhage • Postpartum infection Child: • Perinatal mortality • Large-for-gestational age neonate • Nirth trauma including shoulder dystocia, nerve palsy, bone fracture • Fetal macrosomia • Small-for-gestational age neonate • Low birth weight neonate • Intrauterine growth restriction • Preterm birth • Neonatal hypoglycaemia • Respiratory distress syndrome • Neonatal jaundice • Neonatal hypocalcaemia • Neonatal adiposity • Polycythaemia • Apgar sore < 7 at 5 min • Admission to neonatal intensive care unit or special care nursery
6. Time span of prediction	Complications occurring during pregnancy (obstetric) or affecting the neonate (neonatal), defined per standard definition which is an infant during the first 28 days after birth.
7. Intended moment of using the model	At any time during pregnancy.

### Eligibility criteria

Study selection will be based on pre-determined eligibility criteria framed using the PICOTS system [36] (Table 2). PICOTS is a modification of the established PICO system tailored to the specific requirements of systematic reviews of prediction models with additional consideration for timing (both for the time period of the prediction and the time point at which the prediction model is to be used) and clinical setting [35].

**Table 2:** Eligibility criteria for the systematic review framed using the PICOTS system [36]

	Inclusion criteria	Exclusion criteria
Population	Women with gestational diabetes (any diagnostic criteria).	Women with pre-gestational diabetes (type 1 and type 2 diabetes).
Index	Development or external validation of a prognostic prediction model for women (e.g. prediction model for women with gestational diabetes to predict pregnancy complications)	Diagnostic prediction models (e.g. prediction model for the diagnosis of GDM)
Comparator	Not applicable	
Outcomes (primary)	Obstetric: - Hypertensive disorders of pregnancy - Caesarean delivery Neonatal: - Large-for-gestational age (LGA) - Composite of perinatal (fetal and neonatal) mortality or serious morbidity* (e.g. birth trauma, shoulder dystocia, bone fracture or nerve palsy)	
Outcomes (secondary)	Obstetric: - Maternal mortality - Placental abruption - Induction of labour - Perineal trauma - Postpartum haemorrhage - Postpartum infection - Instrumental delivery Neonatal: - Perinatal (fetal and neonatal) mortality - Serious morbidity (e.g. birth trauma, shoulder dystocia, bone fracture or nerve palsy) - Fetal macrosomia - Small-for-gestational age (SGA) - Low birth weight - Intrauterine growth restriction (IUGR) - Preterm delivery or premature birth - Neonatal hypoglycaemia - Respiratory distress syndrome - Neonatal jaundice - Neonatal hypocalcaemia - Neonatal adiposity - Neonatal polycythaemia - Apgar score < 7 at 5 min - Admission to neonatal intensive care unit	
Timing	Complications occurring during pregnancy or up to 6 weeks postpartum period (obstetric or maternal) or affecting the neonate (neonatal).	Complications affecting the mother pre-pregnancy or with an onset more than 6 weeks postpartum. Complications affecting the offspring after 28 days of age.
Setting	Prognostic prediction models that are intended to be used by healthcare professionals, in the antenatal clinic setting, at any time during pregnancy intended to inform clinicians’ therapeutic decision-making.	Prognostic prediction models, which are intended to be used before pregnancy (pre-conception) or after childbirth.
Study type	Any study design including primary research (e.g. randomised controlled trial, cohort study, case-control study) or secondary research (e.g. systematic review) that reports on (ii) one or more statistical models, tools or scores with at least two predictors proposed to predict an individual’s risk of a future outcome (prediction modelling studies). Other names for prediction models include prognostic model, prognostic (or prediction) index or rule, risk (or clinical) prediction model and predictive model. Prediction models estimate the risk of experiencing the outcome and may be reported in absolute (absolute probability) or relative (risk score) terms [37]. Prediction modelling studies can be either model development, model validation or a combination.	Editorial comments or letters.

*For studies set in low and middle-income countries perinatal mortality and serious morbidity will be assessed as individual primary outcomes

#### Population

Studies reporting on prediction models proposed for pregnant women with GDM will be considered for inclusion. GDM may have been diagnosed by any criteria. Studies proposing models for pregnant women with pre-gestational diabetes (type 1 and type 2 diabetes) will be excluded.

#### Intervention

Prediction model development studies with and without external validation and external model validation studies with or without model updating will be considered for inclusion if they are intended to inform clinicians’ therapeutic decision making regarding the management of a pregnancy affected by GDM.

#### Outcomes

The included pregnancy complications related to GDM and their prioritisation were aligned with those agreed by consensus of the Cochrane Pregnancy and Childbirth Group responsible for systematic reviews for prevention and treatment of GDM and pre-existing diabetes [31, 32] and drew on published search strategies for similar review questions [20, 38]. The timing and effect measures for each outcome will be as defined by the study’s authors. Complications potentially related to the treatment of GDM such as maternal hypoglycaemia and glycaemic control were not included. GWG was not included as it is also likely to be a predictor of the outcomes of interest.

#### Timing

Included studies need to report on prediction models for complications occurring during pregnancy or the postpartum period or affecting the neonate. The standard definition of neonate will be used, that is, an infant during the first 28 days after birth. Prediction models for complications with onset after this period will be excluded.

#### Setting

Prediction models that are intended to be used by healthcare professionals in the antenatal clinic setting, at any time during pregnancy will be considered for inclusion. Models intended to be used before (pre-conception) or after (post-partum) will be excluded.

#### Types of studies and limits

Any study design including primary research (e.g. randomised controlled trial or observational study) that reports on one or more statistical models, tools or scores with at least two predictors proposed to predict an individual’s risk of a future outcome (prediction modelling studies) will be considered for inclusion. Other names for prediction models include prognostic model, prognostic (or prediction) index or rule, risk (or clinical) prediction model and predictive model. Risk predictions are usually expressed in absolute terms as a probability, i.e. 0 to 100% (but can be relative (risk score) [39]. Any identified and relevant review articles will be used to identify eligible primary studies.

Studies will be limited to those conducted in humans by applying The Cochrane Group’s filter for Humans not Animals filter [40]. There will be no limits on the year of publication hence included articles will be from all years in the MEDLINE (from 1946) and Embase (from 1947) databases. No restriction to language of publication will be applied.

### Search methods for identification of studies

#### Information sources

The following electronic databases will be searched to identify eligible studies:

- Ovid MEDLINE(R) and Epub Ahead of Print, In-Process and Other Non-Indexed Citations, Daily and Versions(R) on OvidSP (from 1946 to present)
- Embase Classic+Embase on OvidSP (from 1947 to present)

The reference list of included studies will be hand searched for additional potentially relevant citations.

#### Search strategy

A sensitive search strategy, based on the eligibility criteria and combining subject indexing terms (i.e. MeSH) and free-text search terms in the title and abstract fields, will be developed for MEDLINE using the OvidSP platform. The search strategy, specifically, subject indexing terms will be translated appropriately for Embase.

The search strategy will be iteratively developed and refined with the assistance of clinical advisors (HJT, JAB and GS), a medical librarian and an evidence synthesis expert. The final search strategy will combine concepts related to prognostic factors and prediction modelling studies, GDM and pregnancy complications. The updated version of a validated filter for prediction modelling studies published by Geersing and colleagues [41] (based on the original published by Ingui and colleagues [42]) will be used. For GDM, a search strategy published in a peer-reviewed systematic review of treatments for this condition will be used [43]. These two concepts will be combined with a bespoke search strategy for pregnancy complications related to GDM defined as the outcomes of interest in the eligibility criteria (Table 2). The draft search strategy is provided in Additional file 1: Table S1.

A backward citation search will be conducted on all model development studies. All retrieved studies will be reviewed to identify all relevant external validation studies.

## Data collection and analysis

### Data management

Retrieved studies will be imported into Endnote reference manager software. (Version X8.2, Clarivate Analytics, Philadelphia, USA. Available at https://endnote.com/) Duplicate records will be identified and excluded using a systematic, rigorous and reproducible method utilising a sequential combination of fields including author, year, title, journal and pages [44]. Covidence systematic review software will be used to manage records throughout the review. (Veritas Health Innovation, Melbourne, Australia. Available at http://www.covidence.org).

### Selection process

Two reviewers (SDC, LAW) will independently screen the titles and abstracts of every article retrieved by the search strategy according to the selection criteria (Table 2). Full text of the articles will be retrieved for further assessment if the information given suggests that the study meets the selection criteria or if there is any doubt regarding eligibility of the article based on the information given in the title and abstract. Any disagreement will be resolved by discussion to reach a consensus and consultation with an advisor (HJT) if required. For publications in languages other than English portions of the title, abstract and full-text article will be translated as necessary. A record of all retrieved studies will be maintained and reasons for exclusion documented.

### Data extraction

Two reviewers (SDC, LAW) will independently extract the data from the included studies using a standardised electronic form developed with reference to the CHecklist for critical Appraisal and data extraction for systematic Reviews of prediction Modelling Studies (CHARMS) [30]. Variables for which data will be sought will include information on objective, source of data, participants, outcome(s) to be predicted, candidate predictors, sample size, missing data, model development, model performance (discrimination, calibration and measures of case-mix variation), results including final multivariable models and interpretation of presented models [30]. Data on diagnostic approach for GDM used (diagnostic criteria, testing procedures and screening policies), whether population was treated and treatment type will also be extracted. Any disagreement will be resolved by discussion to reach a consensus and consultation with an advisor (HJT) if required. Missing data will be obtained from the authors wherever possible; if insufficient information is obtained the study will be excluded.

### Critical appraisal

The methodological quality (risk of bias) and relevance (applicability) to the review question of included studies will be systematically assessed using the prediction model risk of bias assessment tool (PROBAST) [35]. This tool is structured around four key domains: participants, predictors, outcome and analysis. Each domain is rated as “high”, “low” or “unclear” risk of bias. Two reviewers (SDC, LAW) will independently evaluate the risk of bias and applicability of each included study. Any disagreement will be resolved by discussion to reach a consensus and consultation with an advisor (HJT) if required. Results will be presented graphically with each study given a rating of low, unclear or high risk for each of the four domains.

### Qualitative data synthesis of prediction models

All extracted data on prediction models from included studies will be tabulated to facilitate comparison of outcomes to be predicted, predictors included in the final model and performance measures [30, 38]. Measures of uncertainty will be reported when published or approximated using published methods [36]. Where reported, classification measures such as sensitivity, specificity, positive predictive value and negative predictive value will also be included [30, 38]. A descriptive analysis of key items will also be presented.

### Quantitative analysis and comparison of the predictive performance of prediction models

The nature of the quantitative analysis will be dependent on the number of prediction models identified in the systematic review and the type of prediction modelling study (i.e. development or validation).

Data will be synthesised by performing meta-analysis by type of prediction modelling study if feasible and identified prediction models are sufficiently homogenous. Clinical homogeneity will be satisfied if the review identifies:

- Multiple validation studies for a common prediction model are identified or
- Multiple development studies where the target population to whom the model applies, outcome to be predicted and intended moment of using the model are considered similar.

### Meta-analysis and investigation of heterogeneity

Where meta-analysis is feasible, performance measures such as discrimination (e.g. concordance (c) statistic or area under the curve) and calibration (e.g. total number of observed to expected events ratio [total O:E ratio]] and calibration slope) will be will be pooled and analysed using a random-effects meta-analysis model to provide estimates of the average performance of the model across the included studies. To estimate the between-study heterogeneity and the 95% confidence intervals for this average performance, the restricted maximum likelihood and the Hartung-Knapp-Sidik-Jonkman methods will be used respectively [36]. Meta-analysis will be conducted with reference to the Meta-analysis of Observational Studies in Epidemiology (MOOSE) group guidelines [45] using the metareg module in Stata (StataCorp).

Heterogeneity in performance measures is anticipated and will likely reflect the heterogeneity of study design and population [36]. The range of potential model performance in a different population in a new validation study will be estimated by calculating an approximate 95% prediction interval [36]. Case-mix variation within each study will be quantified by estimating the standard deviation of the linear predictor [36]. When performance measures or measures of uncertainty have not been reported, they will be approximated where possible using appropriate methods [36]. Statistical homogeneity will be assessed using the *I*^2^ test where *I*^2^ values over 50% indicate moderate to high heterogeneity [46]. Potential sources of heterogeneity will be investigated by undertaking a meta-regression analysis.

#### Analysis of subgroups

Where there are sufficient number of included studies sub-group analyses will be undertaken.

Subgroup analyses will be conducted according to the type of prediction modelling study (development and/or validation), target population to whom the prediction model applies as defined by diagnostic approach to GDM (diagnostic criteria, testing procedures and screening policies), testing procedures or screening policies for GDM used, whether population was treated (yes/no), treatment type, outcome to be predicted, intended moment of using the model and study quality (risk of bias).

#### Sensitivity analysis

Sensitivity analyses will be performed for studies at lower and higher risk of bias to explore the influence of risk of bias on effect size [36].

### Reporting and presentation of findings

Reporting and presentation of results will be guided by the PRISMA statement (preferred reporting items for systematic reviews and meta-analyses) [47]. Relevant recommendations from the TRIPOD statement (transparent reporting of a multivariable prediction model for individual prognosis or diagnosis) [34] will also be considered.

The GRADE approach (grading of recommendations, assessment, development and evaluation) will be applied to determine confidence in estimates [48, 49].

## Discussion

This systematic review will identify all published prognostic prediction models for pregnancy complications in women with GDM. These prognostic prediction models will be comprehensively summarised and their performance compared across different settings and populations with meta-analysis if appropriate.

A prognostic prediction model for GDM that is intended to aid therapeutic decision-making during pregnancy would ideally integrate clinically meaningful and patient aligned outcomes including pregnancy complications affecting mother and baby. Long-term outcomes affecting maternal health such as progression to type 2 diabetes and resultant increased cardiovascular risk for mother and affecting offspring health such as increased risk of childhood obesity for offspring are important. However, they are less likely to have a significant bearing on clinical decision-making during the pregnancy itself. Rather prediction of long-term risks should facilitate the targeting of preventative interventions post-partum.

Prognosis-related research in GDM is a relatively novel field of enquiry. The glucometabolic health and outcomes of a pregnant woman are influenced by a number of clinical factors, including glucose levels, body mass index (BMI), GWG and ethnicity [17–20]. These predictors have been incorporated into prediction models but to date, the focus has been on GDM diagnosis rather than prognosis after GDM diagnosis. Diagnostic prediction models seek to predict the risk of GDM diagnosis based on routinely available clinical parameters with a view to directing screening and/or primary prevention efforts. Multiple diagnostic prediction models have been developed [38], including our own [50], and 12 were recently externally validated and compared head-to-head in a prospective cohort [51].

The utility of diagnostic prediction models for GDM diagnosis is limited in contemporary clinical practice because case-finding based on high-predicted risk of diagnosis has been superseded by universal screening at 24–28 weeks gestation in most guidelines. Furthermore, guidelines are increasingly recommending first-trimester screening for type 2 diabetes in women at high risk, identified by the presence of one or more risk factors [10, 12, 13]. Although widely assumed in routine clinical practice the benefit of early diagnosis and treatment is yet to be demonstrated and thus is currently subject to a randomised controlled trial [52].

This review will make an important contribution to the understanding of the risk of pregnancy complications for women with GDM. Furthermore, it will promote the consideration of the broad continuum of risk related to this condition in routine clinical practice. If this review does not identify any applicable models or applicable models have poor performance and or methodological quality, then these results will provide rationale and guidance for model development and/or updating. Conversely, if this review identifies a prognostic model with high predictive performance, applicability and methodological quality, then such a model could be implemented and would be valuable to clinicians caring for women with GDM. It would allow clinicians to predict an individual’s absolute risk of pregnancy complications. This prediction could also help affected women understand the implications of GDM on their pregnancy and in doing so, promote shared decision-making with clinicians that consider individual risk estimated objectively and systematically. At a health service level, the implementation of such prediction models would support a personalised risk-stratified model-of-care, which would ultimately better direct finite health resources to women at high-risk and most likely to benefit from intervention.

As such, this systematic review serves as the foundation for a body of work to develop, validate, implement and evaluate the impact of a prognostic prediction model for pregnancy complications related to GDM across the four themes of the PROGRESS prognosis research framework [53]. Theoretical prediction models not implemented into clinical practice are a waste of research effort and resources. Hence, the authors’ will adopt a model aggregation approach to develop a meta-model which optimally captures prior knowledge by combining model validation and updating of the existing prediction models identified in this review [54]. The performance of the meta-model will be compared to existing single models identified in this review, facilitating the selection of the prognostic prediction model most suitable for application into clinical practice. This model will become the subject of implementation research. An impact study will be designed and conducted to compare a model-of-care based on the directive use of the prediction model to stratify women to targeted interventions based on their risk of pregnancy complications compared to usual care [55]. Such a study would also facilitate evaluation of the implementation of this prognostic model into the model-of-care including its acceptability to clinicians and impact on health service utilisation and costs.

Ultimately, this systematic review is an important step towards developing and implementing personalised risk-stratified models-of-care for GDM. This will allow preventative and therapeutic interventions to be precisely targetted at women most likely to benefit, and sparing expense and harm for those who will not.

## Supplementary information

**Additional file 1:** **Table S1.** Search strategy for MEDLINE. **Figure S1.** Draft PRISMA Flow Diagram for the systematic review process.

**Additional file 2:** PRISMA-P 2015 Checklist.

## Data Availability

Data sharing is not applicable to this article as no datasets were generated or analysed.

## Abbreviations

BMI: Body mass index
CHARMS: CHecklist for critical Appraisal and data extraction for systematic Reviews of prediction Modelling Studies
GDM: Gestational diabetes mellitus
GRADE: Grading of Recommendations, Assessment, Development and Evaluation
GWG: Gestational weight gain
IADPSG: The International Association of Diabetes in Pregnancy Study Groups
MOOSE: Meta-analysis of Observational Studies in Epidemiology
PRISMA: Preferred Reporting Items for Systematic reviews and Meta-analyses
PROGRESS: PROGnosis RESearch Strategy
TRIPOD: Transparent Reporting of a multivariable prediction model for Individual Prognosis Or Diagnosis
