# Supplementary material for: Prognostic prediction models for pregnancy complications in women with gestational diabetes: a protocol for systematic review, critical appraisal and meta-analysis
Source: Syst Rev. 2019 Nov 11;8:270. doi: 10.1186/s13643-019-1151-0 (PMC6844063; doi:10.1186/s13643-019-1151-0)
Supplement: Supplementary file 1 — Additional file 1: Table S1. Search strategy for MEDLINE. Figure S1. Draft PRISMA Flow Diagram for the systematic review process. [file 13643_2019_1151_MOESM1_ESM.pdf]

Additional file 1

Table S1. Draft search strategy for MEDLINE.

Concept	Step	Search Strategy
**Study type = prediction modelling studies**		
Study type = prediction modelling studies (Ingui filter for prediction models(1))	1	Validat$.mp. or Predict$.ti. or Rule$.mp.
	2	(Predict$ and (Outcome$ or Risk$ or Model$)).mp.
	3	((History or Variable$ or Criteria or Scor$ or Characteristic$ or Finding$ or Factor$) and (Predict$ or Model$ or Decision$ or Identif$ or Prognos$)).mp.
	4	Decision$.mp. and ((Model$ or Clinical$).mp. or Logistic Models/)
	5	(Prognostic and (History or Variable$ or Criteria or Scor$ or Characteristic$ or Finding$ or Factor$ or Model$)).mp.
	6	or/1-5
Study type = prediction modelling studies (Addition to Ingui filter proposed by Geersing to improve sensitivity(2))	7	Stratification.mp. or "ROC Curve"/ or Discrimination.mp. or Discriminate.mp. or c-statistic.mp. or "c statistic".mp. or "Area under the curve".mp. or AUC.mp. or Calibration.mp. or Indices.mp. or Algorithm.mp. or Multivariable.mp. [mp=title, abstract, original title, name of substance word, subject heading word, floating sub-heading word, keyword heading word, protocol supplementary concept word, rare disease supplementary concept word, unique identifier, synonyms]
Combination of study type concepts	8	6 or 7
**Population = women with gestational diabetes**		
	9	exp diabetes, gestational/
	10	(gestation* adj4 diabet*).ti,ab.
	11	gdm.ti,ab.
	12	(glucose adj4 (pregnan* or gestation* or prenatal* or antenatal* or pre-natal* or ante-natal* or maternal*)).ti,ab.
	13	or/9-12
**Outcomes = pregnancy complications**		
Hypertensive disorders of pregnancy	14	exp Hypertension, Pregnancy-Induced/
	15	((hypertens* adj3 pregnan*) or gestational hypertens* or pre-eclamp* or preeclamp* or eclamp* or HELLP syndrome).ti,ab.
	16	14 or 15
Perinatal mortality	17	exp Perinatal Mortality/ or Fetal Mortality/ or Perinatal Death/ or exp Fetal Death/
	18	(((perinatal or neonatal or f?etal or intrauterine or intra-uterine) adj (mortalit* or death* or demise or loss)) or stillbirth*).ti,ab.
	19	17 or 18
Large-for-gestational age (LGA)	20	exp Birth Weight/
	21	((Large* adj3 gestation*) or LGA or large birth*).ti,ab.

	22	20 or 21
Perinatal morbidity	23	exp Birth Injuries/ or exp $Dystocia/ or exp Brachial Plexus Neuropathies/ or exp Obstetric Labor Complications/
	24	(birth injur* or ((birth or labo?r or obstetric* or perinatal or delivery) adj4 (trauma* or injur* or complication*1)) or (shoulder adj4 dystocia*) or (fracture*1 adj4 (clavic* or humerus or shoulder*1 or arm*1)) or ((neonat* or infant* or erb* or obstetric or brachial) adj5 (palsy or neuropath*)) or nerve palsy or plexus injury).ti,ab.
	25	23 or 24
Caesarean delivery	26	exp Cesarean Section/
	27	(c?esar* or (delivery adj abdominal) or c-section).ti,ab.
	28	26 or 27
Maternal mortality	29	Maternal Mortality/ or Maternal Death/
	30	((maternal or mother* or woman or women or pregnan* or gestation*) adj2 (mortalit* or death* or dies or died)).ti,ab.
	31	29 or 30
Induction of labour	32	exp Labor, Induced/
	33	(induc* adj3 (labo?r or deliver*)).ti,ab.
	34	32 or 33
Placental abruption	35	Abruptio Placentae/
	36	(placenta* adj2 abrupt*).ti,ab.
	37	35 or 36
Perineal trauma	38	Episiotomy/
	39	((perine* adj2 (trauma or injur* or tear*)) or ((birth or sphincter*) adj (injury or trauma)) or (third-degree adj tear*) or (fourth-degree adj tear*) or episiotom*).ti,ab.
	40	38 or 39
Postpartum haemorrhage	41	Postpartum Hemorrhage/
	42	((postpartum or post-partum) adj1 h?emorrhage).ti,ab.
	43	41 or 42
Neonatal hypoglycaemia	44	hypoglycemia/ and (infan* or neonat* or newborn* or baby or babies).ti,ab.
	45	(((infan* or neonat* or newborn* or baby or babies) and hypoglyc?em*) or (((infan* or neonat* or newborn* or baby or babies) and low) adj2 (glucose or sugar))).ti,ab.
	46	44 or 45
Fetal macrosomia	47	exp Fetal Macrosomia/
	48	macrosomi*.ti,ab.
	49	47 or 48
Small-for-gestational age	50	Infant, Small for Gestational Age/
	51	((small* adj3 gestation*) or SGA).ti,ab.
	52	50 or 51
Low birth weight	53	exp Infant, Low Birth Weight/
	54	(low-birth-weight or low birthweight*).ti,ab.
	55	53 or 54
	56	exp Fetal Growth Retardation/

Intrauterine growth restriction (IUGR)	57	(fetal growth restrict* or fetal growth retard* or intra-uterine growth retard* or intra-uterine growth restrict* or intrauterine growth retard* or intrauterine growth restrict* or IUGR).ti,ab.
	58	56 or 57
Preterm delivery or premature birth	59	exp Obstetric Labor, Premature/ or exp infant, premature/
	60	(((pre-term* or preterm* or premature) adj birth*) or premat*).ti,ab.
	61	59 or 60
Admission to neonatal intensive care unit	62	Intensive Care, Neonatal/ or Intensive Care Units, Neonatal/
	63	(((neonatal or newborn*) adj1 (intensive or unit* or ICU*)) or ((neonatal or special) adj2 nursery) or NICU).ti,ab.
	64	62 or 63
Neonatal respiratory distress syndrome	65	Respiratory Distress Syndrome, Newborn/
	66	((infan* or neonat* or newborn* or baby or babies) and (respiratory distress or dyspn?ea or cyanosis or RDS)).ti,ab.
	67	65 or 66
Neonatal jaundice	68	exp Hyperbilirubinemia, Neonatal/
	69	((infan* or neonat* or newborn* or baby or babies) adj5 (hyperbilirubin* or jaundice)).ti,ab.
	70	68 or 69
Neonatal hypocalcaemia	71	Hypocalcemia/ and (infan* or neonat* or newborn* or baby or babies).ti,ab.
	72	((infan* or neonat* or newborn* or baby or babies) and (hypocalc?emi* or (low adj2 calcium))).ti,ab.
	73	71 or 72
Neonatal adiposity	74	Pediatric obesity/ and (infan* or neonat* or newborn* or baby or babies).ti,ab.
	75	(((infan* or neonat* or newborn* or baby or babies) adj5 (adipos* or skin-fold or skinfold or weight or body mass index or fat or abdominal circumference or obesity or anthropometr*)) or ponderal index).ti,ab.
	76	74 or 75
Postpartum infection	77	Puerperal Infection/
	78	((postpartum* or post-partum* or puerper*) and (infect* or sepsi*)).ti,ab.
	79	77 or 78
Apgar score < seven at 5 minutes	80	Apgar Score/
	81	apgar*.ti,ab.
	82	80 or 81
Neonatal polycythaemia	83	Polycythemia/ and (infan* or neonat* or newborn* or baby or babies).ti,ab.
	84	((infan* or neonat* or newborn* or baby or babies) adj3 polycyth?emia).ti,ab.
	85	83 or 84
Instrumental delivery	86	exp Extraction, Obstetrical/ or exp Delivery Obstetric/ or exp Vacuum Extraction, Obstetrical/

	87	((obstetric* or instrument* or forcep* or vacuum*) adj2 (deliver* or extraction*)).ti,ab.
	88	86 or 87
‘Pregnancy complications’ as a general concept	89	exp Pregnancy Outcome/ or exp Pregnancy Complications/
	90	((pregnan* or perinatal or obstetric* or labo?r or birth or neonatal or maternal) adj3 (outcome* or complication* or adverse)).ti,ab.
	91	89 or 90
**Combination of all pregnancy complication concepts**	92	16 or 19 or 22 or 25 or 28 or 31 or 34 or 37 or 40 or 43 or 46 or 49 or 52 or 55 or 58 or 61 or 64 or 67 or 70 or 73 or 76 or 79 or 82 or 85 or 88 or 91
**Combination of concepts**	93	8 and 13 and 92
**Human filter**	94	exp animals/ not humans/

Figure S1. Draft PRISMA Flow Diagram for the systematic review process.

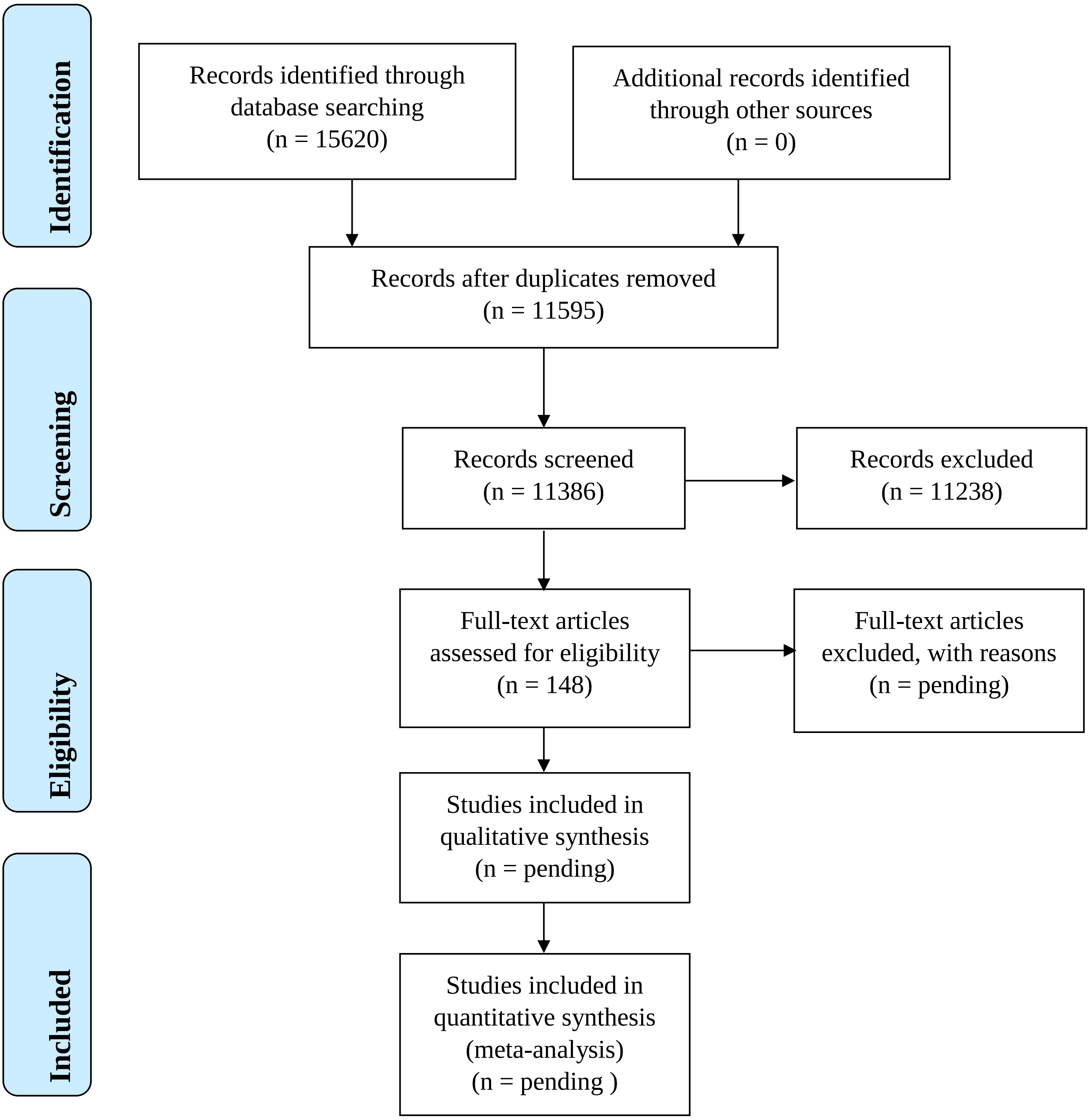

References

1. Ingui BJ, Rogers MA. Searching for clinical prediction rules in MEDLINE. J Am Med Inform Assoc. 2001;8(4):391-7.
2. Geersing GJ, Bouwmeester W, Zuithoff P, Spijker R, Leeflang M, Moons KG. Search filters for finding prognostic and diagnostic prediction studies in Medline to enhance systematic reviews. PLoS One. 2012;7(2):e32844.
